# Evaluating the Role of CBC-Derived Indices in Children with Hashimoto’s Thyroiditis

**DOI:** 10.3390/diagnostics14242834

**Published:** 2024-12-17

**Authors:** Andrei-Ioan Munteanu, Iulius Jugănaru, Delia-Maria Nicoară, Niculina Mang, Raluca Vasilescu, Giorgiana-Flavia Brad, Alexandra-Cristina Scutca, Raluca Asproniu, Lucian-Ioan Cristun, Otilia Mărginean

**Affiliations:** 1 Department XI Pediatrics, Discipline I Pediatrics, ‘Victor Babeş’ University of Medicine and Pharmacy of Timisoara, 300041 Timisoara, Romania; andrei-ioan.munteanu@umft.ro (A.-I.M.); nicoara.delia@umft.ro (D.-M.N.); nina.mang@umft.ro (N.M.); brad.giorgiana@umft.ro (G.-F.B.); scutca.alexandra@umft.ro (A.-C.S.); asproniu.raluca@umft.ro (R.A.); marginean.otilia@umft.ro (O.M.); 2 Department of Pediatrics I, Children’s Emergency Hospital “Louis Turcanu”, 300011 Timisoara, Romania; raluca.bolboase@gmail.com; 3 Research Center for Disturbances of Growth and Development in Children BELIVE, ‘Victor Babeş’ University of Medicine and Pharmacy of Timisoara, 300041 Timisoara, Romania; 4 Ph.D. School Department, ‘Victor Babeş’ University of Medicine and Pharmacy of Timisoara, 300041 Timisoara, Romania; lucian.cristun@umft.ro

**Keywords:** autoimmune thyroiditis, children, NLR, biomarker

## Abstract

**Background/Objectives:** Hashimoto’s thyroiditis (HT) is an autoimmune disorder characterized by chronic inflammation of the thyroid gland. Recent evidence indicates that the inflammation may extend beyond the thyroid. The study aims to explore the potential of complete blood count (CBC)-derived indices as markers of systemic inflammation in HT. **Materials and Methods:** This cross-sectional retrospective study from 1 January 2015, to 31 December 2023 included 147 pediatric HT patients and 144 apparently healthy controls. Thyroid profiles, antibodies, CBC, and protein electrophoresis data were collected from patient records. CBC-derived indices were calculated and compared between the HT and control groups, as well as among HT subgroups. **Results:** The median age of HT patients was 13.6 years (range: 11.2–15.5 years), with 66% being girls. The control group had a similar age and gender distribution, with a median age of 13.7 years (range: 11–15.8 years) and 70.8% girls. Of the HT patients, 50% had subclinical HT, 15% were euthyroid, and 34% had overt thyroid dysfunction. HT patients showed significantly higher neutrophil and lymphocyte counts, as well as all evaluated CBC-derived indices than controls (*p* < 0.001)). These differences were not significant among HT subgroups. Logistic regression indicated a strong association between an elevated neutrophil-to-lymphocyte ratio (NLR) and HT diagnosis (*p* < 0.001), while ROC analysis confirmed NLR as the most accurate CBC-derived marker for distinguishing HT from controls. **Conclusions:** Elevated NLR levels in pediatric HT patients provide additional evidence that inflammation may extend beyond the thyroid gland. These results support the potential of NLR as a reliable and accessible biomarker for evaluating inflammation in Hashimoto’s thyroiditis.

## 1. Introduction

Autoimmune thyroiditis (AIT) is the most common cause of acquired thyroid disease in children [1], with a prevalence estimated at approximately 3% [2]. It is characterized by the progressive destruction of the thyroid gland due to an autoimmune process, leading to gradual thyroid failure [3]. AIT presents in two distinct forms: one associated with the presence of goiter, Hashimoto’s thyroiditis (HT), and the other characterized by the absence of goiter, referred to as atrophic thyroiditis [4]. In children, Hashimoto’s thyroiditis is the most frequent form of AIT, typically presenting during adolescence [4]. Initial presentation can differ based on the extent of immunologic damage [4], with possible thyroid function statuses ranging from complete euthyroidism to subclinical hypothyroidism, overt hypothyroidism, or, in rare instances, subclinical or overt hyperthyroidism (Hashitoxicosis) [4,5,6,7,8]. At the time of diagnosis, most children are either euthyroid or exhibit subclinical hypothyroidism [6,7].

Although classified as an organ-specific autoimmune disease [9], evidence suggests that the inflammation associated with Hashimoto’s thyroiditis may extend beyond the thyroid gland [10]. Recent studies indicate that Hashimoto’s thyroiditis can induce systemic cellular inflammation [11,12], even in individuals with normal thyroid function [13,14,15]. Systemic inflammation is commonly assessed using markers such as C-reactive protein (CRP) and erythrocyte sedimentation rate (ESR) [16]. However, in addition to stimulating protein responses, inflammation drives the overproduction and release of myeloid cells and platelets from the bone marrow into the bloodstream and sites of inflammation, often at the expense of other cell lineages [17]. This leads to changes in the numerical distribution of different types of blood cells in the periphery [18]. As a result, considerable research has focused on exploring hematological indices derived from CBC parameters as alternative markers for detecting and monitoring inflammatory processes [19,20,21]. Such indices are the neutrophil-to-lymphocyte ratio (NLR) [22], the platelet-to-lymphocyte ratio (PLR) [23], the systemic immune-inflammation index (SII) [24], and the system inflammation response index (SIRI) [25], among others. These indices have been suggested as diagnostic and prognostic biomarkers for various diseases with inflammatory or autoimmune components [26,27,28,29,30]. Regarding thyroid disorders, CBC-derived indices have been investigated concerning their potential use in neoplastic disorders [31,32], thyroid nodules [33,34,35], goiter [36], and thyroiditis [37,38]. Most studies regarding these indices in Hashimoto’s thyroiditis have focused on the adult population [39,40,41].

Our study evaluates the relationship between CBC-derived inflammatory biomarkers and their use as markers in Hashimoto’s thyroiditis in pediatric patients.

## 2. Materials and Methods

### 2.1. Study Design and Patient Recruitment

**Figure 1:**
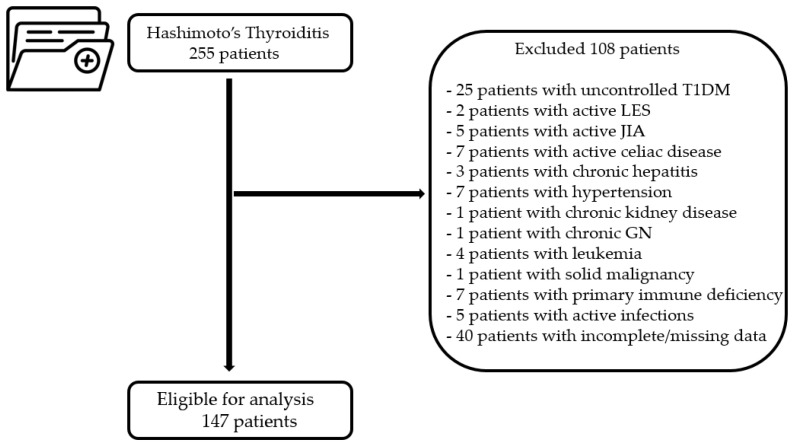
Schematic representation of the HT patient selection process. T1DM, type 1 diabetes mellitus; LES, systemic lupus erythematosus; JIA, juvenile idiopathic arthritis; GN, glomerulonephritis.

This retrospective, cross-sectional study was conducted at the Children’s Emergency Hospital “Louis Turcanu” Timișoara, Romania. The medical records of 255 patients diagnosed with Hashimoto’s thyroiditis between 1 January 2015, and 31 December 2023 were retrieved and systematically evaluated. The inclusion criteria were an age of 18 years or younger and a diagnosis of Hashimoto’s thyroiditis. Patients with concurrent active autoimmune diseases, hepatic, cardiovascular, renal, hematologic, or myeloproliferative disorders, as well as those with active infections or incomplete laboratory investigations, were excluded from the study, as illustrated in Figure 1.

The diagnosis of HT was established with a combination of clinical features, elevated serum antibody titers (anti-thyroid peroxidase or anti-thyroglobulin levels) and characteristic findings on thyroid ultrasound [6]. Furthermore, based on hormonal alteration on blood essays, HT patients were categorized into the following subgroups: euthyroid HT, subclinical hypothyroid HT, overt hypothyroid HT, and hyperthyroid HT.

Control patients were selected from children aged 18 years or less, admitted for minor surgical procedures (e.g., hernia repair) who had no underlying systemic illness and whose preoperative screening evaluations showed normal CBC and acute phase reactants.

### 2.2. Ethical Considerations

The study protocol received approval from the hospital’s ethics committee (decision number: 12241/3 July 2024), and complied with the guidelines from the Declaration of Helsinki 1975 (revised in 2013). The requirement for patient consent for the secondary use of data was waived by the Ethics Committee for Research of the “Victor Babes” University of Medicine and Pharmacy, Timisoara, in consideration of the study’s retrospective design and the utilization of anonymized datasets.

### 2.3. Data Collection

Demographic characteristics and laboratory data were retrieved for each patient from electronic records and medical charts.

Hematological investigations were performed with a Sysmex XS800i analyzer using impedance spectroscopy, flow cytometry, and hydrodynamic focusing (direct current detection method). Reagents for these analyses were provided by Sysmex Corp. (Kobe, Japan). A complete blood count (CBC) was obtained from 1 mL of peripheral venous blood, collected in an EDTA (sodium calcium formate) tube.

The following thyroid parameters were measured using immunochemistry with enzyme chemiluminescence immunoassay (ECLIA) on a Roche Cobas 601 autoanalyzer (Roche Diagnostics, Mannheim, Germany): free triiodothyronine (fT3), free thyroxine (fT4), and serum thyrotropin (TSH). IgG class serum thyroglobulin antibodies (anti-TGAb) and thyroid peroxidase antibodies (anti-TPO) were measured using the Enzyme-Linked Fluorescent Assay technique on a VIDAS^®^ 3 analyzer (BioMérieux SA, Marcy-l’Étoile, France). Serum protein electrophoresis was performed using agarose gel (Hydragel 30 β1–β2, Sebia, Lisses, France) on a Hydrasys 2 semi-automatic analyzer (Sebia, Lisses, France).

CBC-derived inflammatory indices were calculated using the following formulas: neutrophil-to-lymphocyte ratio (NLR: neutrophil/lymphocyte), platelet-to-lymphocyte ratio (PLR: thrombocytes/lymphocytes), systemic inflammatory index (SII: neutrophils × thrombocytes/lymphocytes), and systemic inflammatory response index (SIRI: neutrophils × monocytes/lymphocytes).

### 2.4. Statistics

Statistical analysis was performed with Statistical Package for Social Sciences software v28.0 (SPSS; IBM Corp: Armonk, NY, USA). Distribution and variance homogeneity were evaluated using the Kolmogorov–Smirnov test. Medians and interquartile ranges (IQR) served to present continuous variables with a non-normal distribution, while categorical variables were described using frequencies and percentages. Comparisons between the HT group and controls were performed using the Mann–Whitney U test, and the Kruskal–Wallis H test analyzed differences across HT subgroups. In addition, relationships between the CBC-derived indices and HT diagnosis were evaluated using Spearman’s correlation analysis, multiple logistic regression, and analysis of receiver operating characteristic (ROC) curves.

A *p*-value (two-tailed) < 0.05 was considered statistically significant.

## 3. Results

### 3.1. General Characteristics of the Study Population

After applying the inclusion and exclusion criteria (Figure 1), the final study population included 147 children diagnosed with Hashimoto’s thyroiditis and 144 age—and gender-matched controls. The median age of the entire HT study population was 13.6 [interquartile range (IQR): 11.2, 15.5] years, with a female predominance similar to that of the control group. Demographic and laboratory data are detailed in Table 1.

### 3.2. Comparison of CBC Parameters and Indices Across HT Subgroups

According to the thyroid profile, half of the patients had subclinical HT, with relatively similar percentages having euthyroidism (15%), overt hypothyroidism (17%), and hyperthyroidism (17%). There were no significant differences in age or gender between individual subgroups (*p* = 0.120 and *p* = 0.739, respectively). Demographic and laboratory data of HT subgroups are summarized in Table 2.

Approximately half of the patients with HT had no comorbidities. One-third of the cohort was classified as obese, while 18% presented with a stature deficit. Twelve patients were identified with type 1 diabetes mellitus, maintaining stable glycemic control (HbA1c < 6.5%). Additionally, 23% of HT patients exhibited a range of other comorbidities, including Down syndrome (*n* = 5), selective IgA deficiency (*n* = 3), Turner syndrome (*n* = 3), and epilepsy (*n* = 3). Other less frequent comorbidities included 21-hydroxylase deficiency (*n* = 2), and single cases of progesterone insufficiency, hyperstature, respiratory arrhythmia, schizophrenia, hypertrichosis, and precocious puberty.

Regarding CBC parameters, HT patients had significantly higher levels of neutrophils and lymphocytes than the control group. Furthermore, all CBC-derived indices investigated were significantly more elevated in HT patients (*p* < 0.001).

As expected, several laboratory parameters showed significant variations. Specifically, Anti-TPO antibody levels were significantly different across subgroups (*p* = 0.007), with the highest levels observed in the hypothyroid subgroup. Similarly, TSH, fT3, and fT4 levels exhibited significant differences among the subgroups (*p* < 0.001), reflecting the thyroid status variations.

Regarding CBC parameters, there were no significant differences in the absolute counts of neutrophils, lymphocytes, and platelets across HT subgroups. However, monocyte levels were lower in euthyroid HT patients than in other subgroups (*p* = 0.044). Furthermore, none of the evaluated CBC-derived indices differed across the subgroups. Regarding globulin fractions, the only statistically significant difference was noted in beta globulins, which were significantly higher in hyperthyroid HT patients.

### 3.3. Correlation Analysis of Hematological Parameters and Indices with HT Diagnosis

**Figure 2:**
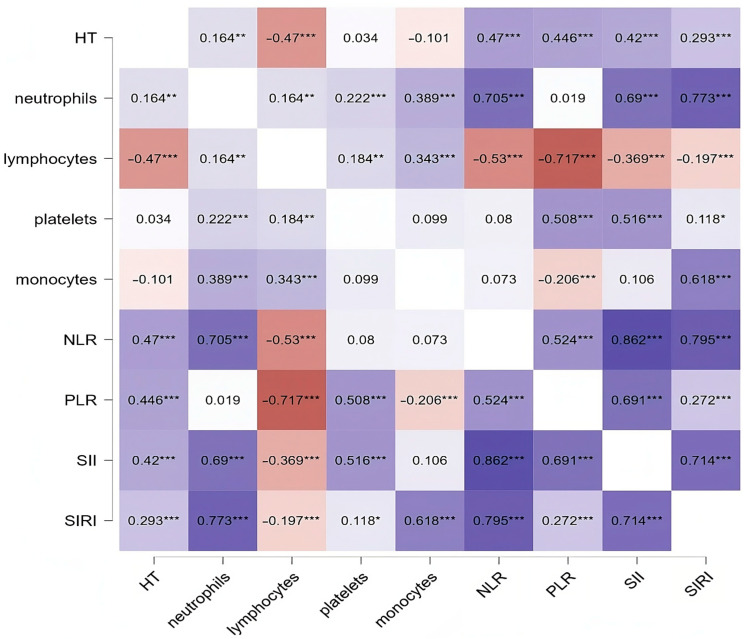
Correlation between CBC parameters and indices and HT diagnosis. HT, Hashimoto’s thyroiditis; NLR, neutrophil/lymphocyte ratio; PLR, platelet/lymphocyte ratio; SII, systemic inflammation index; SIRI, systemic inflammation response index. The significance levels were marked as *** *p* < 0.001, ** *p* < 0.01, and * *p* < 0.05. The accompanying figure employs a color gradient to depict significance levels, with darker shades representing more substantial associations and lighter shades indicating lesser degrees of statistical significance.

The relationship between CBC parameters, derived indices, and HT diagnosis was assessed using Spearman correlation analysis (Figure 2). Significant negative correlations were observed between HT diagnosis and lymphocyte count (ρ = −0.470). Additionally, all four evaluated indices correlated with HT diagnosis, with the strongest correlations for NLR (ρ = 0.470) and PLR (ρ = 0.446).

### 3.4. Relationship Between NLR and HT Diagnosis

Furthermore, multiple logistic regression examined the relationship between NLR and HT diagnosis among the entire study population. This analysis demonstrated that an elevated NLR was strongly associated with an HT diagnosis, even after adjusting for age and gender, as shown in Table 3.

In addition, we evaluated the accuracy of NLR in identifying HT patients by comparing it with several CBC-derived indices, as seen in Table 4.

**Figure 3:**
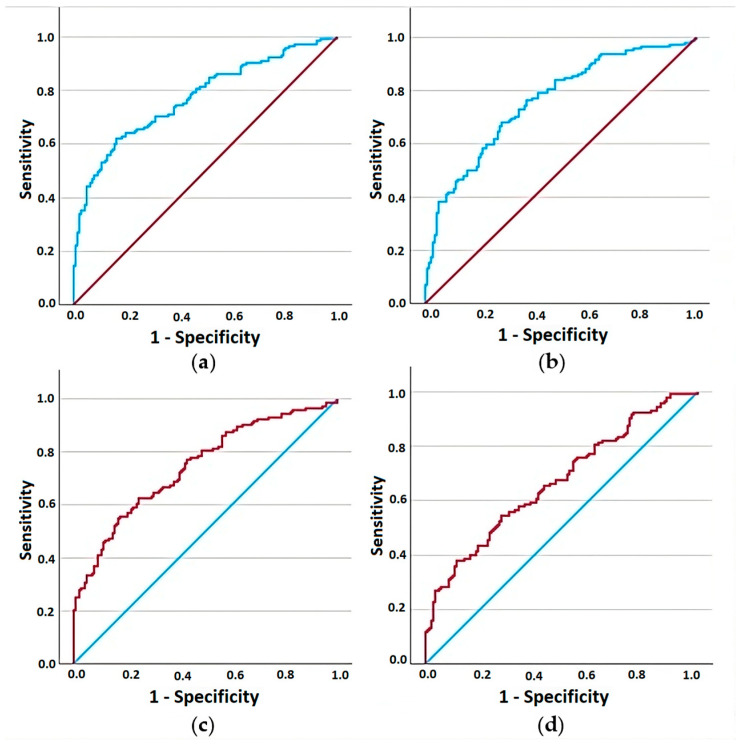
Receiver operating characteristic curve analysis for evaluating the performance of (**a**) NLR, (**b**) PLR, (**c**) SII, and (**d**) SIRI in discriminating HT patients from controls.

As illustrated in Table 4 and Figure 3, the most significant accuracy for HT diagnosis was displayed by NLR (77% sensitivity and 56% specificity) and PLR (76% sensitivity and 63% specificity), followed closely by the SII (74% sensitivity and 58% specificity).

## 4. Discussion

Autoimmune thyroid diseases originate from a combination of genetic, hormonal, and environmental, whose complex interactions trigger inappropriate immune responses against thyroid tissue [42,43]. Several environmental factors have been investigated, including iodine excess, selenium deficiency, smoking, and industrial pollutants. Infectious agents, immunomodulating drugs, and stress are also among the factors that favor disease development [44]. Genetic factors also contribute to the progression of these conditions, as evidenced by linkage and association studies that have identified several key genes, including both thyroid-specific and immune-regulatory genes [45,46,47]. In addition to genetic variations, post-transcriptional and post-translational modifications may also contribute to the onset of autoimmunity, such as the influence of mRNA translation on protein binding [48]. A hallmark biochemical feature of autoimmune thyroid diseases is the presence of thyroid autoantibodies (TAbs) in the serum, which target two major thyroid antigens: thyroid peroxidase (TPO) and thyroglobulin (Tg). These autoantibodies, TPOAb and TgAb, are of the immunoglobulin G (IgG) class and exhibit high affinity for their respective antigens. Unlike TgAb, TPOAb can activate the complement system, leading to thyroid cell damage via antibody-dependent cytotoxicity [43,49].

Although the etiology and systemic involvement of HT remain incompletely understood [10], its pathogenesis is driven by an inflammatory response initiated by the autoimmune activation of T lymphocytes [39], leading to lymphocyte infiltration and atrophy [50]. As in many inflammatory and autoimmune disorders, neutrophils, lymphocytes, and platelets play a central role, exerting their effects through various cytokines [43,51,52]. These cytokines, in turn, perpetuate a vicious cycle of immune cell activation, ultimately leading to tissue destruction [53]. This inflammatory process can lead to alterations in the distribution and functionality of these immune cells, as reflected in changes to the CBC [18,54,55]. Consequently, numerous studies have focused on evaluating the role of various CBC-derived indices in inflammatory and autoimmune diseases, such as NLR and PLR, as well as more recent indices like SII and SIRI [7,56,57,58,59]. However, given the inconsistent correlation with acute phase response markers observed across studies, Bilge et al. hypothesized that these indices likely reflect a distinct aspect of immune dysregulation, separate from the acute phase response [10].

Neutrophilia and lymphopenia are the most commonly observed changes in complete blood counts in inflammatory diseases [60]. Consequently, most research on CBC-derived indices has focused on NLR, which has been studied in neoplastic, cardiovascular, and autoimmune diseases, as well as in various thyroid pathologies. According to Liu et al., an elevated preoperative NLR was linked to larger tumor sizes and a heightened relapse risk in patients with differentiated thyroid cancer [61]. Similarly, Gong et al. reported that preoperative NLR was strongly correlated with the stage of papillary thyroid carcinoma [62]. A recent study by Topuz et al. proposed that the NLR could be an effective measure for monitoring treatment response in individuals with subacute thyroiditis [63]. The significance of NLR has also been studied in Hashimoto’s thyroiditis, with several adult studies reporting higher NLR values in HT patients compared to controls [10,39,40,41,64]. In contrast, others have found similar NLR values between the two groups [12]. Our study also revealed significantly higher NLR values in HT patients (1.766, IRQ: 1.31, 2.37) than in controls (1.211, IQR: 1.00, 1.48). This value is similar to that reported by Erinc et al. and lower than the ones reported by Onalan et al. (2.43 ± 0.94), Bilge et al. (2.29 ± 0.65) and Aktas et al. (2.1, IQR: 1.3−5.8) [10,39,40,64]. The difference might be attributed to the fact that these are all adult studies, and NLR increases with age [65]. When NLR was compared across HT subgroups [39,64], no statistically significant differences were found, aligning with our results. Compared to the adult population, studies regarding CBC-derived indices in children with HT are scarce. The two studies we identified, by Elmaoğulları et al. [66] and Kırkgöz et al. [67], both published in national academic journals, reported similar NLR values in HT patients and controls. A possible reason for the discrepancy between our findings and those of the studies mentioned above could be differences in study design. The first study included only euthyroid pediatric HT patients, while the second study did not specify the proportion of euthyroid versus hypothyroid patients and included only children older than 8 years. The correlation between NLR and thyroid function tests has been studied, but results remain inconsistent. Some studies identified a weak positive correlation between NLR and thyroid autoantibodies [41,64], while others reported no such relationship [10,39]. In pediatric studies, Elmaoğulları et al. found a weak correlation between NLR and TSH, whereas Kırkgöz et al. found no correlations with thyroid autoantibodies. Our findings align with the latter observation [66,67].

Beyan et al. were the first to report a case of reactive thrombocytosis associated with Hashimoto’s thyroiditis, which gradually normalized with thyroid hormone replacement therapy [68]. Inflammatory factors can stimulate thrombopoietin production, leading to increased platelet levels [69]. At the same time, elevated tumor necrosis factor-α causes platelet hyperreactivity and transcriptional reprogramming of megakaryocytes, further promoting platelet activation, adhesion, and aggregation with leukocytes [70]. Hence, CBC-derived indices involving platelets, such as the PLR and SII, have also been studied. The utility of these indices has been investigated in the context of thyroid cancer [71,72], subacute thyroiditis [73,74,75], and Graves’ disease [76,77]. In the case of Hashimoto’s thyroiditis, several studies have reported significantly elevated PLR [10,41,64,78] and SII [64], while others found similar values compared to controls [12]. Our findings regarding PLR align with those of the pediatric studies by Kırkgöz et al. [67] and Elmaoğulları et al. [66], which also observed higher values in HT patients compared to controls, with similar average values (133.46, IQR: 108.6−156.6, 126.7 +/− 83.7, and 138.6 +/− 44.28, respectively). Our study also found significantly elevated SII values in HT patients, unlike Kırkgöz et al. [67].

The literature contains few accounts regarding the role of monocytes/macrophages in autoimmune thyroiditis [79]. The Systemic Inflammatory Response Index, a composite marker of systemic inflammation, combines data from monocytes, neutrophils, lymphocytes, and platelets [80]. A study involving data from 1641 subjects in the National Health and Nutrition Examination Survey (NHANES) 2009–2012 revealed a significant positive correlation between SIRI and FT4 [81]. Furthermore, Xie et al. revealed higher SIRI values in B-Raf proto-oncogene, serine/threonine kinase (BRAF) mutation in patients with papillary thyroid carcinoma [82], while He et al. integrated SIRI in a formula to distinguish SAT patients with thyrotoxicosis from Graves disease patients [73]. We did not find any studies investigating the use of SIRI in Hashimoto’s thyroiditis. In our evaluation, SIRI levels were significantly higher in HT patients than in controls. However, ROC analysis showed that SIRI had the lowest AUC among the four CBC-derived indices for distinguishing HT patients from controls (0.669 for SIRI, compared to 0.744 for SII, 0.759 for PLR, and 0.773 for NLR).

The results of our study have to be interpreted in the context of several limitations. First, the retrospective design of the study could lead to selection bias. This type of data collection does not assure that all eligible patients are evaluated since missing data in medical records cannot be prevented. Second, because of its cross sectional design, results of our study do not aid in patient follow-up, since understanding the relationship between CBC derived indices and disease evolution requires a longitudinal study. Further studies examining changes in CBC-derived parameters over the course of Hashimoto’s thyroiditis could provide valuable insights into their potential as biomarkers for disease progression and therapeutic response. Third, the moderate sample size and focus on a single center may limit the generalizability of our results. All the limitations mentioned above could be addressed in a prospective longitudinal study.

## 5. Conclusions

The results of this study suggest that pediatric patients with Hashimoto’s thyroiditis may exhibit elevated NLR levels, indicating an inflammatory response that could involve both the thyroid gland and systemic inflammation. While these findings highlight the potential of NLR as a biomarker for assessing inflammation in Hashimoto’s thyroiditis, further research is necessary to validate its reliability and establish its clinical utility in diverse patient populations.

## Figures and Tables

**Table 1:** Comparison of demographic and laboratory data between patients with Hashimoto’s Thyroiditis and the Control Group.

Variables	HT Group (*n* = 147)	Control GROUP (*n* = 144)	*p*- Value
Demographic characteristics			
Age (years)	13.6 (11.2, 15.5)	13.7 (11, 15.8)	0.663
Females % (*n*)	97 (66)	102 (70.8)	0.605
Comorbidities			
None % (*n*)	71 (48.3)	n/a	n/a
Obesity % (*n*)	33 (22.4)	n/a	n/a
Stature deficit % (*n*)	18 (12.2)	n/a	n/a
Type 1 diabetes % (*n*)	12 (8.16)	n/a	n/a
Others % (*n*)	23 (15.6)	n/a	n/a
Thyroid status			
Anti-TPO ab (kU/L)	419.7 (74.4, 1000)	n/a	n/a
Anti-TG ab (kU/L)	64.6 (13.5, 273)	n/a	n/a
TSH (mU/L)	4.78 (2.43, 9.05)	n/a	n/a
fT3 (ng/L)	6.24 (5.27, 7.05)	n/a	n/a
fT4 (pmol/L)	15.01 (13.2, 18.6)	n/a	n/a
CBC parameters			
Neutrophils (×10^3^ mm^3^)	3.71 (2.90, 5.07)	3.36 (2.80, 4.22)	**0.004**
Lymphocytes (×10^3^ mm^3^)	2.15 (1.76, 2.52)	2.79 (2.41, 3.23)	**<0.001**
Monocytes (×10^3^ mm^3^)	0.57 (0.47, 0.71)	0.60 (0.51, 0.75)	0.089
Platelets (×10^6^ mm^3^)	276 (240, 328)	282 (234, 324)	0.510
CBC derived indices			
NLR	1.766 (1.31, 2.37)	1.211 (1.00, 1.48)	**<0.001**
PLR	133.46 (108.6, 156.6)	99.2 (79.2, 123.3)	**<0.001**
SII	488.3 (374.9, 693.2)	339.4 (253.7, 435.6)	**<0.001**
SIRI	1.006 (0.699, 1.532)	0.747 (0.571, 1.011)	**<0.001**

Abbreviation: HT, Hashimoto’s thyroiditis; Anti-TPO ab: anti-thyroid peroxidase antibody; Anti-TG ab: anti-thyroglobulin antibody; TSH: thyroid stimulating hormone; fT3, free triiodothyronine; fT4, free thyroxine; NLR, neutrophil/lymphocyte ratio; PLR, platelet/lymphocyte ratio; SII, systemic inflammation index; SIRI, systemic inflammation response index. Statistically significant differences, indicated by a *p*-value of less than 0.05, are highlighted in bold.

**Table 2:** General Characteristics of the HT subgroups according to thyroid status.

Variables	Euthyroid HT (*n* = 23)	Subclinical HT (*n* = 74)	Hypothyroid HT (*n* = 25)	Hyperthyroid HT (*n* = 25)	*p*- Value
Age (years)	14.9 (11.5, 15.8)	13.1 (11.2, 14.6)	14.5 (11, 15.4)	13.9 (11.8, 16.5)	0.120
Females % (*n*)	16 (69.5)	47 (63.5)	17 (68)	17 (68)	0.739
Anti-TPO ab (kU/L)	53.9 (26.6, 398.5)	523 (133, 1000)	1000 (230, 1000)	356 (97.5, 864)	**0.007**
Anti-TG ab (kU/L)	72.3 (21.9, 189.8)	117 (12.2, 302)	51.2 (9.27, 110)	24.9 (12.2, 30.5)	0.320
TSH (mU/L)	2.04 (1.23, 2.65)	5.27 (4.30, 8.29)	39.3 (15.7, 100)	0.005 (0.005, 0.21)	**<0.001**
fT3 (ng/L)	6.25 (5.74, 6.67)	6.03 (5.33, 6.71)	4.80 (4.23, 6.39)	11.4 (8.10, 15.9)	**<0.001**
fT4 (pmol/L)	15.43 (14.7, 17.8)	14.8 (13.8, 16.9)	9.88 (7.66, 11.67)	26.8 (21.2, 41.1)	**<0.001**
Neutrophils (×10^3^ mm^3^)	3.15 (2.85, 4.14)	3.98 (3.05, 5.34	3.64 (2.76, 6.29)	3.95 (2.75, 4.87)	0.345
Lymphocytes (×10^3^ mm^3^)	1.99 (1.76, 2.34)	2.16 (1.75, 2.53)	1.97 (1.81, 2.65)	2.25 (1.76, 2.45)	0.736
Monocytes (×10^3^ mm^3^)	0.49 (0.46, 0.58)	0.58 (0.49, 0.72)	0.57 (0.46, 0.71)	0.59 (0.50, 0.82)	**0.044**
Platelets (×10^6^ mm^3^)	284 (247, 301)	274 (243, 331)	254 (226, 285)	303 (245, 348)	0.299
NLR	1.68 (1.27, 2.05)	1.83 (1.39, 2.50)	1.65 (1.23, 2.45)	1.70 (1.51, 2.35)	0.809
PLR	140 (122, 163)	132 (109, 156)	124.3 (99.5, 149)	148 (111, 163)	0.311
SII	466 (380, 591)	493 (378, 704)	436 (326, 773)	506 (377, 837)	0.742
SIRI	0.76 (0.60, 1.03)	0.99 (0.71, 1.58)	1.04 (0.68, 1.56)	1.27 (0.83, 1.56)	0.138
Alpha1 globulins %	2.10 (2.00, 2.40)	2.20 (2.10, 2.38)	2.20 (2.05, 2.45)	2.50 (2.30, 2.75)	0.111
Alpha2 globulins %	9.30 (8.80, 10.1)	9.95 (9.50, 10.6)	10.5 (9.95, 10.9)	10.1 (9.82, 11.1)	0.092
Beta globulins %	9.92 (8.60, 9.85)	9.75 (9.03, 11)	9.45 (8.53, 10.7)	10.40 (9.80, 10.9)	**0.031**
Gamma globulins %	12.5 (11.7, 14.2)	12.4 (11.7, 13.9)	12.5 (10.9, 15.3)	12.8 (12.2, 14.6)	0.951

Abbreviations: HT, Hashimoto’s thyroiditis; Anti-TPO ab: anti-thyroid peroxidase antibody; Anti-TG ab: anti-thyroglobulin antibody; TSH: thyroid stimulating hormone; fT3, free triiodothyronine; fT4, free thyroxine; NLR, neutrophil/lymphocyte ratio; PLR, platelet/lymphocyte ratio; SII, systemic inflammation index; SIRI, systemic inflammation response index. Statistically significant differences, indicated by a *p*-value of less than 0.05, are highlighted in bold.

**Table 3:** Multiple Regression Analysis.

Variables	OR (95%CI)	*p*-Value
Age	0.933 (−0.170, 0.031)	0.174
Gender	0.636 (−1.063, 0.157)	0.146
NLR	9.932 (1.636, 2.955)	**<0.001**

Abbreviations: OR, odds ratio; CI, confidence interval; NLR, neutrophil/lymphocyte ratio. Statistically significant differences, indicated by a *p*-value of less than 0.05, are highlighted in bold.

**Table 4:** Comparison of CBC-derived indices in discriminating HT from controls.

Variable	AUC	SE	95%CI	Sensitivity	Specificity	Cut-Off	*p*-Value
NLR	0.773	0.027	0.719–0.826	0.772	0.556	1.28	**<0.001**
PLR	0.759	0.028	0.704–0.814	0.757	0.627	108	**<0.001**
SII	0.744	0.029	0.688–0.801	0.743	0.577	375	**<0.001**
SIRI	0.669	0.032	0.607–0.731	0.662	0.543	0.79	**<0.001**

Abbreviations: NLR, Neutrophil-to-lymphocyte ratio; PLR, Platelet-to-lymphocyte ratio; SII—Systemic immune-inflammation index; SIRI, Systemic immune-inflammation response; AUC—Area under the curve; SE—Standard Error; 95%CI—95% Confidence Interval. Statistically significant differences, indicated by a *p*-value of less than 0.05, are highlighted in bold.

## Data Availability

The data are not publicly available due to reasons of privacy.
